# Detection of Hemizygous Chromosomal Copy Number Variants in Williams-Beuren Syndrome (WBS) by Duplex Quantitative PCR Array: An Unusual Type of WBS Genetic Defect

**Published:** 2008-09

**Authors:** Godfrey E. Etokebe, Stefan Axelsson, Niels Henrik Svaerd, Kari Storhaug, Zlatko Dembić

**Affiliations:** 1 *Immunology Laboratory, Department of Oral Biology, University of Oslo, Oslo, Norway;*; 2 *TAKO-Centre, Lovisenberg Diakonale Hospital, Oslo, Norway*

**Keywords:** human, chromosome 7, deletion, copy number variants, genome

## Abstract

We have developed a dual probe quantitative PCR (qPCR) mini array enabling a more accurate analysis of the relationship between copy number variants (CNVs) and other genomic features in specific areas. We used it to map hemizygous microdeletion on human chromosome 7 around the elastin gene (ELN), which is the molecular basis of the Williams-Beuren syndrome (WBS). In two WBS patients, the haploid content of the elastin gene was ascertained previously by the fluorescence in-situ hybridization (FISH). Our dual-color qPCR assay used this information to normalize for DNA content in all tests. We mapped the extent of the deleted area using 10 loci spanning over 4 Mb. A border region containing the GTF2I gene, usually deleted in most cases, was found about 10 times amplified in both patients, suggesting an unusual type of the WBS genetic defect. This 10-WBS-loci-specific qPCR assay could be an affirmative diagnostic tool alternative to FISH. Due to low cost, it could be used as a screening test that would not only facilitate research on CNVs, but also allow early diagnosis of the disease, as well-timed diagnosis would benefit WBS children with earlier proper health-care measures.

## INTRODUCTION

The Williams-Beuren syndrome (WBS) is a complex multisystemic developmental disorder caused by hemizygous microdeletion around the elastin gene on chromosome 7. The WBS is characterized by a unique phenotype that typically includes dysmorphic cranio-facial features, supravalvular aortic stenosis (SVAS), hypertension, mental retardation, premature aging of the skin, infantile hypercalcemia, tooth anomalies, and an unusual set of cognitive and behavioral profiles (1-5). The clinical diagnosis is usually confirmed by fluorescent *in situ* hybridization (FISH) analysis showing hemizygous complement of the elastin gene.

**Figure 1:**
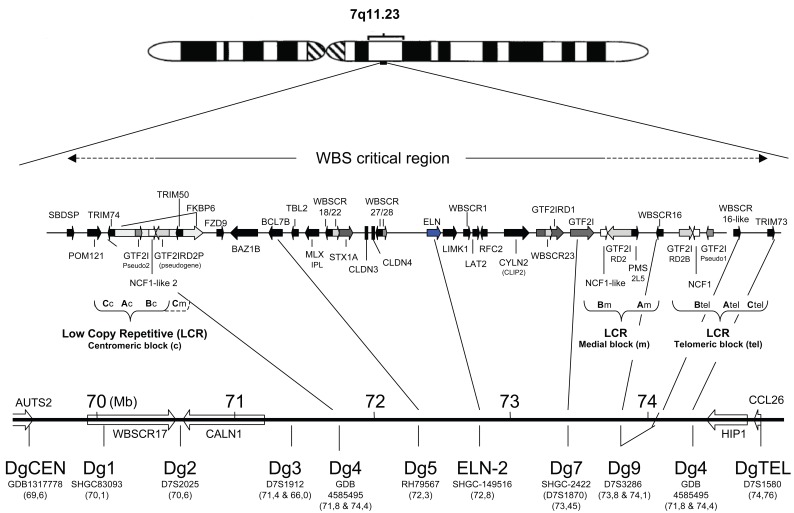
Schematic representation of genetic loci on chromosome 7 used for WBS specific double qPCR assays. The qPCR loci used for making the WS-specific array of qPCR assays are listed at the bottom, together with their precise position from the beginning of the chromosome and STS references. Positions were according to numbering from CRA_TCAGchr7v2 database. The map is according to information from the NCBI database. ELN= the elastin gene.

The commonly deleted area on the chromosome 7 is designated the Williams-Beuren syndrome critical region (WBSCR) and includes over 35 genes, some without known function (Fig. 1). While hemizygosity of the elastin gene is correlated with SVAS, hemideletion of other WBSCR genes is likely to be a contributing factor to diversity of patients’ phenotypes. The most common deletion is about 1.55 Mb in size, and a larger one (about 1.84 Mb) occurs in about 4% of the cases. Less than 1% was reported for other abnormalities (6). However, these reports address only the cases in North America. It seems that the other unusual types of deletions are more common in other parts of the world. In the WBS critical region, deletion borders contain low copy repetitive sequences (LCRs) that share about 95% sequence homology. In the Figure 1, three LCRs were found in the WBSCR: the centromeric (LCRc), medial (LCRm) and telomeric (LCRt). The LCRs contain three different blocks of repetitive sequences (A, B and C), which are mixed between them (resulting with different order and/or orientation in each LCR), and contain several genes including "Neutrophil cytosol factor 1" (NCF1), "general transcription factor II, i" (GTF2I), and "GTF2I repeat domain containing 2" (GTF2IRD2) genes and their look-alikes (Fig. 1). Apparently, this structure fuels genetic instability of this region, with the most common cause for deletion being unequal meiotic crossover (6). The borders for the common WBS deletion of 1.55 Mb were found to be variable within this area due to a large transitional potential of chromosomal breakpoints (up to 143 Kb) (6).

Another contributing factor to WBS like symptoms is the hemizygous inversion of the WBSCR, but not all carriers are symptomatic (7). Inversions of the WBSCR in unaffected parents predispose for WBS in children caused by hemideletion (8). Different types of inversions exist, and despite the lack of statistical evidence so far, as the inversions are difficult to detect, it seems reasonable to assume that they could generate variability in WBS deletions apart from the most commonly observed ones. Recently, a wide variety of deletions ranging between 0.25 and 2.5 Mb in length was reported using quantitative PCR approach (9).

However, it is still unclear to what level various genotypes play a role in the generation of diverse phenotypes of the disease.

Advances in genomic screening technologies such as microchip are revealing structural changes in individual genomes due to copy number variations, inversions, deletions or nucleotide polymorphisms (for a review see (10)). The areas of structural variants differ to a great extent, the smaller the area the more difficult for precise mapping. Thus, the microarray technology could only coarsely estimate the WBS deleted region, and such analysis is always dependent on more reliable qPCR analyses. Similarly, the ELN FISH method is simple, but not so specific when it comes to mapping the deletion area. Here, we wished to complement the research in this field that could also benefit affected individuals providing an earlier diagnosis, as well as to search for genes that can affect dental malformation or agenesis. We developed a 10-locus duplex qPCR assay spanning over 4.5 Mb around WBSCR to detect hemizygous deletions on chromosome 7.

## MATERIALS AND METHODS

### Patients and Controls

Criteria for clinical diagnosis of WBS were previously reported (11). Diagnosis was supported by biomedical assessment of patients in which the tests described in this manuscript provided a part. This work is a pilot study to provide feasibility of the method to embark on a larger genotype/phenotype analysis of craniofacial developmental aberrations. We collected blood samples from two WBS patients at the TAKO-Centre, Lovisenberg Diakonale Hospital, The control DNA included persons not affected by this syndrome, used in studies as described previously (12, 13). All persons provided oral and written informed consent.

### The DNA analysis

DNA was isolated from frozen blood using DNA isolation kit according to manufacturer’s protocol. The control DNA was from the sample #4035 described previously in several reports (12, 13).

The tests were done by Taqman real time PCR assay using MX4000 or MX3005 PCR machines and software (Stratagene, San Diego, USA). The primers and probes for the WBSCR region were designated WS-Dg1-5, WS-Dg7 and WS-Dg9; the controls were WS-CEN, WS-TEL and WS-ELN (centromeric, telomeric and elastin). The reporter dyes were 5’ FAM, except for the WS-ELN probe, which was labeled with HEX. All probes had zero fluorescence quencher at the 3’ end. The double qPCR array kit consisted of a set of WS-ELN-2 primers and probe (HEX) supplemented with one of the above-mentioned sets of primers and FAM-labeled probes.

All assays were done in triplicates, and the software provided by the PCR equipment manufacturer calculated statistics.

We used the following sequences for primers and probes:

The WS-DgCEN qPCR test detects DNA sequence tagged site (STS) GDB: 1317778 (locus AUTS2; Gen-Bank acc. no.: AC_000068; UniSTS: 3517). The assembly map of chromosome 7 from the Center for Applied Genomics at the Hospital for Sick Children in Toronto (annotation of CRA_TCAGchr7v2) places the primers and probes at about 69.57 Mb (Fig. 1). The primers and reporter probe were:

- Forward (DgCen1-fw): 5’-GCTATCTTCAGGTCTCTCCAAACAG (69.565692..668);
- Reverse (DgCen1-rv): 5’-GCATGTGAAGAAGGCAGCTTT (69.565616..636);
- Reporter (DgCen1-pb): 5’-*FAM*-TGGAACAATAACACCAGCGAGATGGGA (69.565665..639).

The WS-Dg1 qPCR test defines STS DNA sequence SHGC83093 (GenBank acc. no.: AC_000068; UniSTS: 184651) located at about 70.38 Mb (Fig. 1) on chromosome 7 (CRA_TCAGchr7v2). The primers and reporter probes for the WS-Dg1 were:

- Forward (Dg1-fw): 5’-GGAGATAGAGACCCCATGTATTGACT (70.379116..091);
- Reverse (Dg1-rv): 5’-GAAACTTGGTGAGTGCCTTCTGT (70.379036..058);
- Reporter (Dg1-pb): 5’-*FAM*-TGGTAGCTCCAGTGGAAATGCCTGCT (70.379089..064).

The WS-Dg2 qPCR test detects STS D7S2025 (Gen-Bank acc. no.: AC_000068; UniSTS: 38585), which is located at about 70.58 Mb on chromosome 7 (CRA_TCAGchr7v2). The primers and reporter probes for the WS-Dg2 were:

- Forward (Dg2-fw): 5’-GTCATAGGGACAGATTCCTCATGA (70.580143..166);
- Reverse (Dg2-rv): 5’-GCATGAACTAATAAAGTAACAATTCACTCA (70.580222..193),
- Reporter (Dg2-pb): 5’-*FAM*-TGACTCGGTGCCCTCCCTG (70.580168..186).

The WS-Dg3 qPCR test identifies sequence close to D7S1912 sequence (Gen-Bank acc. no.: AC_000068; UniSTS: 17311), which occurs at two sites (Fig. 1) about 66.02 and 71.39 Mb on chromosome 7 (CRA_TCAGchr7v2). The primers and reporter probes for the WS-Dg3 were:

- Forward (Dg3-fw): 5’-CATTCTCTCTGTCGTCTTCTGTCTCT (occurs twice: 66.025310..335; 71.389079..054 Mb);
- Reverse (Dg3-rv): 5’-GACCATGAACACCATCAAGTGAA (occurs at 2 sites: 66.025390..368; 71.388999..021 Mb);
- Reporter (Dg3-pb): 5’-*FAM*-CTTCTGAGATTGCCACGTGCAA (occurs at 2 sites: 66.025339..360 and 71.389050..029).

The WS-Dg4 qPCR test defines sequence GDB: 4585495, which occurs at three loci (Fig. 1): tripartite motif-containing 74 (TRIM74), TRIM50, and TRIM73 (GenBank acc. no.: AC_000068). On chromosome 7 (CRA_TCAGchr7v2) the reporter sequence Dg4-pb (22-mer) occurs at three sites, similarly as the reverse primer. However, since the Dg4-forward primer sequence occurs at the first (centromeric, TRIM74) and the last (telomeric, TRIM73) sites, the recognition by this assay is limited to the latter two loci (Fig. 1). The primers and reporter probes for the WS-Dg4 were:

- Forward (Dg4-fw): 5’-CCTCTATTGCCCTCTTTAAGGTGTT (the first 4 nucleotides are not listed in the current database; sequence #5-25 occurs at 2 sites on chromosome 7: 71.766871..851 and 74.363921..941; it differs additionally at position 19 from the sequence at 72.069068..048);
- Reverse (Dg4-rv): 5’-CCCATCAATCGAGCATCTCA (occurs at 3 sites: 71.766804..823; 72.069001..020, and 74.363988..969);
- Reporter (Dg4-pb): 5’-*FAM*-CCGTTGCCTCCTGTTTATTGAG (occurs at 3 sites: 71.766849..828, 72.069046..025 and 74.363943..964).

The WS-Dg5 qPCR test defines RH79567 sequence at locus BCL7B (B-cell CLL/lymphoma 7B; GenBank accession: AC_000068; UniSTS: 92014), at about 72.28 Mb (Fig. 1) on chromosome 7 (CRA_TCAGchr7v2). The primers and reporter probe were:

- Forward (Dg5-fw): 5’-GGCAGGGATGCTGGAATG (72.284001-..018);
- Reverse (Dg5-rv): 5’-GCCCTCAGCACACACATCTG (72.284071..052);
- Reporter (Dg5-pb):5’-*FAM*-CAGGTAGAGGTGAGAACAAAGCTGCGTGT (72.284020..048).

The WS-ELN-2 qPCR test detects STS sequence (SHGC-149516) in the last intron of the elastin gene (Gen-Bank accession: AC005056; UniSTS: 177051) at about 72.78 Mb (Fig. 1) on chromosome 7 (CRA_TCAGchr7v2). The primers and probe were:

- Forward (C2EL-fw; DgCtrl2_ELNgenefw): 5’-GAAGCCTTCCTGGATTTCTCAA (72.778989 ..968; [this sequence occurs also on chr 1]);
- Reverse (C2EL-rv; DgCtrl2_ELNgenerv); 5’-TGCAATGATGAAAGAAGCAGACA (72.778915..937);
- Reporter (C2EL-pb; DgCtrl2_ELNgenepbH); 5’-*HEX*-CTCCTTCTGGCCACCCCCAACC (72.778965..944).

The WS-Dg7 primers and probe were selected from 387-bp-long DNA sequence SHGC-2422 (locus GTF2I; GenBank accession: G10931; UniSTS: 37563) located at about 73.46 Mb on chromosome 7 (CRA_TCAGchr7v2), adjacent to D7S2714 and D7S1870. The primers and probe were:

- Forward (Dg7d-fw): 5’-GCAGGCCCTCCCATCAC (three sites on chromosome 7: 73.455950..934; 86.235358..342; and 122.446747..731; single sites on chr 5, 9, 13 and 16, double sites on chr 1, 6, 8, 14, 15 and X, three sites on chr 11, five sites on chromosomes 2, 3 and 12, and six sites on chr 4);
- Reverse (Dg7d-rv): 5’-TGCTTCTGGTAGTGTTGGAGACA (chr 7: 73.455889..911);
- Reporter (Dg7d-pb): 5’-*FAM*-ACAGCTGTGCCTGGTTGGCA (chr 7: 73.455932..913).

The WS-Dg9 qPCR assay defines two D7S3286 loci (in the WBSCR16 and WBSCR16-like genes; GenBank acc. no.: AC_000068; UniSTS: 254185), located about 73.79 and 74.1 Mb (Fig. 1) on chromosome 7 (CRA_TCAGchr7v2). The primers and reporter probe were:

- Forward (Dg9-fw): 5’-TCCTGGCAAGGGTCTTTGAG (at 73.791169..150 & 74.121373..392);
- Reverse (Dg9-rv): 5’-AGGCTGCGTCCCAAGTCA (at 73.791103..120 & 74.121439..422);
- Reporter (Dg9-pb): 5’-*FAM*-CACAGCCACTGCCCCTGCTTGG (at 73.791148..127 and 74.121394..415).

The WS-DgTEL qPCR test defines STS D7S1580 (GenBank accession: G00254; UniSTS: 39633) located between CCL26 and CCL24 genes (about 74.76 Mb) on chromosome 7 (CRA_TCAGchr7v2). The oligonucleotide set for the WS-DgTEL test comprised the following primers and probe:

- Forward (DgTel2-fw): 5’-AGGACTCCCCCCAAACATG (at 74.762421..439);
- Reverse (DgTel2-rv): 5’-TGTGGTCAGGGAGGGTCTTG (at 74.762494..477);
- Reporter (DgTel2-pb): 5’-*FAM*-CCAGTTTTGGCTTAACTTGGCTAC (at 74.762442..465).

Reactions contained about 25 ng of genomic DNA, and were performed in a final volume of 20 μl, using conditions for Taqman PCR as instructed by the manufacturer (ABI). Briefly, samples were incubated for 10 minutes at 95 deg C to activate the enzyme, and then followed by 40 cycles of 15 seconds at 94 deg C, and 45 seconds at 60 deg C steps. The fluorescence, measured after each cycle, was analyzed with the latest version of MXpro software (Stratagene).

## RESULTS

The STS database at the NCI/NCBI GenBank was searched for unique sequences that are present around the elastin gene in the Williams-Beuren syndrome critical region (WBSCR) on human chromosome 7. We initially selected 12; ten of which were within the WBSCR and two that were just a few Mb outside this area, altogether spanning over 4 Mb of DNA (Fig. 1). The primers and probes for qPCR were designed using Taqman technology, as it obviates the need for identification of amplicons. The fluorescent probes hybridize to specific PCR products supplying the proof for amplifying the right DNA fragments, as determined by the increase of free fluorescent label after each cycle. According to the human genome database (NCBI), our WBS specific qPCR array could detect unique STSs in the WBSCR, except for the WS-Dg3, WS-Dg4 and WS-Dg-9 sets. The WS-Dg3 detects a duplicon (D7S1912) on chromosome 7, one between WS-Dg2 and WS-Dg4 (at 71.1 Mb), and the other 4 Mb centromeric to the WS-DgCEN probe (Fig. 1). The WS-Dg4 assay would detect only two (at 71.8 and 74.4 Mb) of three possible sites that this probe could identify in C blocks of the LCRs (Fig. 1), probably due to lack of forward-primer homologous sequence in medial C block (at 72.1 Mb; see materials and methods for explanation; not shown). The WS-Dg-9 set detects two replicons (D7S3286) in the A blocks of medial and telomeric LCRs (Fig. 1). Three probes (WS-ELN-2, WS-Dg5 and WS-Dg7) detected single copy DNA sequences (in haploid genome) located in the WBS critical region. Outside the WBSCR, four probes (WS-DgCEN, WS-Dg1, WS-Dg2 and WS-DgTel) detected also single copy sequences (Fig. 1).

**Figure 2:**
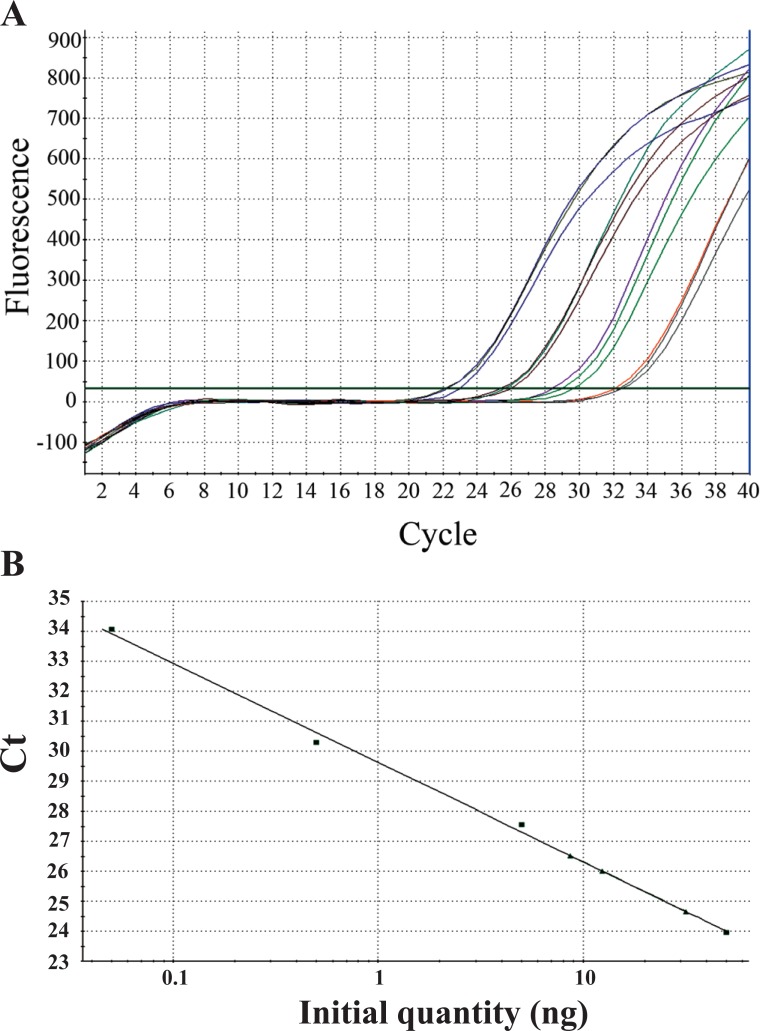
A single qPCR assay for the elastin gene using WS-ELN-2 probe: Amplification plots (qPCR) and the standard curve. A) The graph represents HEX fluorescence levels during the 40-cycle amplification of the elastin gene with specific qPCR. The initial DNA quantity per sample was between 50 and 0.05 ng, as ascertained by the spectrophotometer. The MX4000/MXPro software automatically calculates threshold cycle (Ct) using its algorithm. Values on the graph are linear, indicating good sensitivity within this range of detection of genomic DNA (0.05 ng - 50 ng) in order to calculate the elastin gene copy number; B) The standard curve for the WS-ELN-2 probe, using control DNA in dilutions as in A (filled squares). The triangles depict values of DNA samples taken from two WBS patients and a single control individual, predicted by their Ct value. The position on the curve represents a certain copy number of this probe. Comparison between a patient and a control samples yields relative copy number per genome. The patients’ samples have half the copy number value of the elastin gene than the control sample. Thus the patients have haploid content of the elastin gene, which was corroborated by the in situ chromosomal hybridization (data not shown) during the initial diagnostic process.

We first tested the WS-ELN-2 assay. To generate the standard curve for the elastin gene qPCR we used four tenfold dilutions of control DNA, all in triplicates (Fig. 2A). The intensity of fluorescence (in combination with the fidelity of real time PCR amplification) was measured in order to calculate Ct values and initial DNA amount [ng] per tube using the software provided by the manufacturer. The DNA amount (previously determined by the spectrophotometer) correlated linearly to DNA concentrations calculated by the software (Fig. 2B). Using this standard curve, we measured DNA content of the control subject and two patients in a single dilution assay. The results for two patients varied between 9 and 13 ng per tube, despite an effort to equalize the input by spectrophotometer. This is likely due to a pipetting error, but it could be due to variations in DNA isolation procedure or simply an unknown biological variation. The control sample (having similar DNA input) showed about 30 ng per tube, which is more than a double of both patients' amount. The FISH indicated the haploid content of the elastin gene in both patients (data not shown). Thus, this difference in DNA quantity resembles the haploid or diploid content of the elastin gene in patients or control DNA samples, respectively.

Next, a single-probe qPCR results using control DNA showed that each of the FAM-labeled WS-Dg1,2,3,4,5,7,9, CEN and -TEL assays had similar linear correlation with DNA concentration (data not shown).

**Figure 3:**
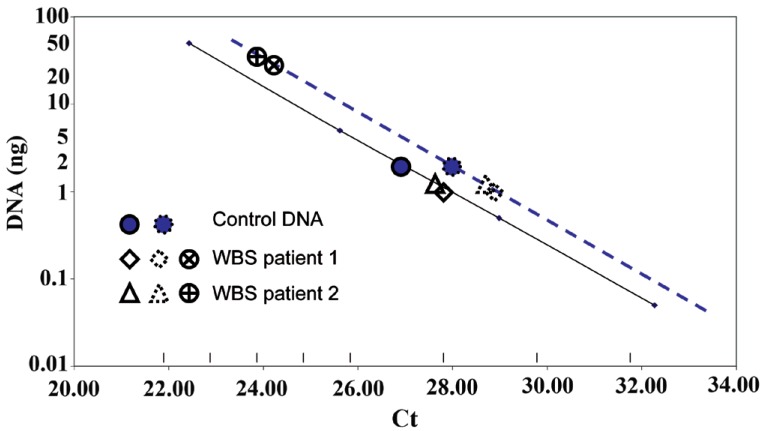
A schematic representation of qPCR assays to detect hemizygous chromosomal microdeletion. The lines represent standard curves for all probes, each used in combination with a single elastin gene probe ELN (dual probe qPCR assays). The ELN probe standard curve is depicted with a full line, and WS-Dg1,2,3,4,5,7, and 9 or WS-DgCEN and WS-DgTEL probes are represented with a dotted line. The points (control, patient 1 and 2 DNA) were single measurements (in triplicates). The elastin probe (HEX; filled squares or triangles) was used in combination with each of the WS-Dg probes (FAM; open squares or triangles): The filled circles represent control (full line) or patient (dotted line) samples. The circles with a cross inside signify patients’ DNAs analyzed with the WBS-Dg7 (FAM) qPCR assay. These circles indicate that patients’ samples have lower Ct compared to the star-like filled circle of the control one, indicating over 10-fold amplification of the genomic area detected by the WBS-Dg7 probe. Thus, patients have over 10 times higher copy number variant of the WBS-Dg7 area than the control individual.

WBS specific qPCR assays consisted of a double-probe array of assays. Each assay had a set of primers and one FAM-labeled probe mixed with the elastin gene specific set of primers and its HEX-labeled probe (WS-ELN-2). Mixing two different sets of primers and probes in making an array of single-tube assays did not affect Ct linearity with DNA concentration in all possible cases, as shown in Fig. 3. Such conditions allowed the measurement of DNA content for each WS-Dg locus in samples from WBS patients (Fig. 3). The fidelity of qPCR amplifications was between 80 to 110% (Table 1).

**Table 1:** The specific dual quantitative PCR analyses of the WBSCR

**A.**	**WS-ELN-2**^a^			**WS-DgCEN**^b^			**CEN/ELN-2**
Sample	C_T_ (s.d.)	PCR eff.^c^	ng (s.d.)	C_T_ (s.d.)	PCR eff.^c^	ng (s.d.)	[ng/ng]
*Patient1*	25.92 (0.07)	106%	11.27 (0.57)	25.93 (0.07)	107%	22.50 (1.13)	2.00
*Patient2*	25.92 (0.04)	- _"_ -	11.28 (0.35)	25.86 (0.08)	- _"_ -	23.67 (1.40)	2.10
*Control DNA*	24.88 (0.04)	- _"_ -	23.90 (0.73)	25.82 (0.06)	- _"_ -	24.39 (1.02)	1.02^d^
**B.**	**WS-ELN-2**^a^			**WS-Dg1**^b^			**Dg1/ELN-2**
Sample	C_T_ (s.d.)	PCR eff.^c^	ng (st. dev.)	C_T_ (s.d.)	PCR eff.^c^	ng (st. dev.)	[ng/ng]
*Patient1*	24.90 (0.03)	88%	11.80 (0.20)	24.80 (0.22)	97%	28.50 (4.37)	2.42
*Patient2*	25.25 (0.13)	- _"_ -	9.47 (0.75)	25.11 (0.02)	- _"_ -	22.80 (2.65)	2.41
*Control DNA*	23.90 (0.04)	- _"_ -	22.10 (0.54)	24.83 (0.10)	- _"_ -	27.70 (1.92)	1.25^d^
**C.**	**WS-ELN-2**^a^			**WS-Dg2**^b^			**Dg2/ELN-2**
Sample	C_T_ (s.d.)	PCR eff.^c^	[ng/ng]	C_T_ (s.d.)	PCR eff.^c^	ng (st. dev.)	[ng/ng]
*Patient1*	27.71 (0.04)	103%	5.89 (0.17)	28.00 (0.12)	92%	9.83 (0.76)	1.67
*Patient2*	26.76 (0.07)	- _"_ -	11.54 (0.54)	26.07 (0.03)	- _"_ -	34.50 (0.69)	2.99
*Control DNA*	25.62 (0.01)	- _"_ -	25.89 (0.27)	26.68 (0.01)	- _"_ -	23.20 (0.12)	0.89^d^
**D.**	**WS-ELN-2**^a^			**WS-Dg3**^b^			**Dg3/ELN-2**
Sample	C_T_ (s.d.)	PCR eff.^c^	ng (s.d.)	C_T_ (s.d.)	PCR eff.^c^	ng (s.d.)	[ng/ng]
*Patient1*	24.62 (0.02)	98%	11.19 (0.16)	24.94 (0.06)	98%	20.38 (0.78)	1.82
*Patient2*	24.92 (0.06)	- _"_ -	9.23 (0.39)	25.17 (0.04)	- _"_ -	17.46 (0.42)	1.89
*Control DNA*	23.59 (0.06)	- _"_ -	22.96 (0.99)	24.83 (0.05)	- _"_ -	22.01 (0.73)	0.96^d^
**E.**	**WS-ELN-2**^a^			**WS-Dg4**^b^			**Dg4/ELN-2**
Sample	C_T_ (s.d.)	PCR eff.^c^	ng (s.d.)	C_T_ (s.d.)	PCR eff.^c^	ng (s.d.)	[ng/ng]
*Patient1*	23.38 (0.09)	106%	15.20 (0.99)	23.32 (0.11)	105%	25.64 (2.03)	1.69
*Patient2*	23.79 (0.10)	- _"_ -	11.35 (0.82)	23.69 (0.04)	- _"_ -	19.95 (0.57)	1.76
*Control DNA*	22.85 (0.04)	- _"_ -	22.36 (0.68)	23.58 (0.21)	- _"_ -	21.77 (3.29)	0.97^d^
**F.**	**WS-ELN-2**^a^			**WS-Dg5**^b^			**Dg5/ELN-2**
Sample	ng (s.d.)	C_T_ (s.d.)	PCR eff.^c^	ng (s.d.)	C_T_ (s.d.)	PCR eff.^c^	[ng/ng]
*Patient1*	27.76 (0.15)	97%	8.25 (0.83)	32.79 (0.29)	86%	2.85 (0.53)	0.35
*Patient2*	27.44 (0.14)	- _"_ -	10.36 (0.95)	30.73 (0.21)	- _"_ -	10.13 (1.34)	0.98
*Control DNA*	26.41 (0.06)	- _"_ -	20.77 (0.86)	29.84 (0.12)	- _"_ -	17.53 (1.30)	0.84^d^
**G.**	**WS-ELN-2**^a^			**WS-Dg7**^b^			**Dg7/ELN-2**
Sample	C_T_ (s.d.)	PCR eff.^c^	ng (s.d.)	C_T_ (s.d.)	PCR eff.^c^	ng (s.d.)	[ng/ng]
*Patient1*	26.91 (0.02)	112%	9.62	23.79 (0.22)	108%	81.50	8.5
*Patient2*	26.94 (0.05)	- _"_ -	9.38	23.57 (0.24)	- _"_ -	95.90	10.2
*Control DNA*	25.46 (0.06)	- _"_ -	27.98	25.40 (0.29)	- _"_ -	24.54	0.88
**H.**	**WS-ELN-2**^a^			**WS-Dg9**^b^			**Dg9 / ELN-2**
Sample	C_T_ (s.d.)	PCR eff.^c^	ng (s.d.)	C_T_ (s.d.)	PCR eff.^c^	ng (s.d.)	[ng/ng]
*Patient1*	25.44 (0.05)	109%	12.74 (0.47)	23.81 (0.11)	114%	22.25 (1.83)	1.75
*Patient2*	25.72 (0.08)	- _"_ -	10.36 (0.59)	23.84 (0.12)	- _"_ -	21.76 (2.00)	2.10
*Control DNA*	25.50 (0.06)	- _"_ -	25.35 (1.12)	23.56 (0.18)	- _"_ -	26.99 (3.85)	1.06d
**I.**	**WS-ELN-2**^a^			**WS-DgTEL**^b^			**TEL/ELN-2**
Sample	C_T_ (s.d.)	PCR eff.^c^	ng (s.d.)	C_T_ (s.d.)	PCR eff.^c^	ng (s.d.)	[ng/ng]
*Patient1*	25.27 (0.09)	81%	11.30 (0.62)	27.31 (0.15)	80%	18.34 (1.57)	1.62
*Patient2*	24.88 (0.00)	- _"_ -	14.27 (0.95)	26.88 (0.21)	- _"_ -	23.63 (2.90)	1.66
*Control DNA*	24.29 (0.06)	- _"_ -	20.15 (0.62)	26.96 (0.10)	- _"_ -	22.47 (1.32)	1.12^d^

a The elastin gene probe;

b The WBS diagnostic probes;

c The efficiency of the PCR reaction;

d The values represent accuracy of each dual PCR test. In dual PCR tests the ratio between calculated DNA contents of various WBS probes and the elastin gene probe should be theoretically 1.00 in the control DNA samples as they have a diploid genome.

In dual probe qPCR assays, the difference between DNA concentration measured using the elastin gene qPCR and that from another locus indicated the copy number difference between them. It is expressed in terms of multiples of the elastin gene copy number (Table 1). Since the former was known to be present in a single copy of patients’ DNA (as determined by FISH; data not shown), the copy number of the elastin gene locus was arbitrarily set to equal 1, in order to make Table 2. The corollary of this setup is that it normalizes for DNA input in all dual assays, thus avoiding pipetting error or biological variability in final calculations. The relative values are shown in Table 1, and rounded up as to estimate probable copy numbers of indicated loci are summarized in Table 2.

**Table 2:** Estimated copy number of loci spread over 4 Mb region encompassing the WBSCR

Loci	Control^a^	Patient 1^a^	Patient 2^a^

*DgCEN*	2	2	2
*Dg1*	2	2	2
*Dg2*	2	2	3
*Dg3*	4	4	4
*Dg4*	4 (6)	4 (5)	4 (5)
*Dg5*	2	1	1
*Elastin (ELN-2)*	2	1	1
*Dg7*	1	>8	>10
*Dg9*	4	4	4
*DgTEL*	2	2	2

The copy numbers of listed loci in two WBS patients and controls were estimated from measurements by dual qPCR in Table 1.

a Copy number content per genome.

In all dual assays, the ELN-2 assay consistently showed that the elastin gene copy number was always double in control than patients’ samples. The WS-Dg1 - 4 sequences were all similar between patients and the control, indicating that their copy number was unaffected in patients. The exception is perhaps WS-Dg4, which recognizes four (C block specific; Fig. 1) sites, but could be recognizing 6 loci, provided a single-nucleotide mismatch in forward primer did not suppress its amplification (see Materials and methods). If so, then a third (0.33) Ct difference would indicate a loss of a single site and could possibly explain our findings (Table 1). The WS-Dg5 locus (BCL7B) was found to be in the same copy number as the elastin gene in one, and probably in both patients; otherwise it is hard to explain *two-thirds* of a single copy number in the other patient (Table 1). The copy number of sequences detected by the WS-Dg9 probe appeared to be 4 in all tested individuals. Likewise, the control probes from telomeric (WS-DgTEL) and centromeric (WS-DgCEN) genetic areas were in diploid number (Table 2). Interestingly, WS-Dg7 sequence was found hugely amplified in both patients. About 8-10 fold increase in genomes was due to unknown reasons (Tables 1 and 2). However, it is tempting to speculate that these replicons might have substituted the deleted part of the chromosome 7. The latter could have been preferentially selected during mitosis preserving perhaps the original (undeleted) length of the chromosome.

In conclusion, the extent of the hemideletion can be approximately narrowed to an area between STS sequences detected by WS-Dg4 and WS-Dg7 probes (Fig. 1). This genetic area spans minimally 0.5 Mb to maximally 1.7 Mb in length (Fig. 1), which is in agreement with previous reports on hemideletion in the WBSCR (6). The most unusual feature is the large copy number (8-10 times) of the genetic region around the WS-Dg7-probe that detects 9th intron of the GTF2I gene.

## DISCUSSION

The goal of our research is to correlate genotype and phenotype of the Williams-Beuren syndrome, especially in persons with dental abnormalities. As a first step, we developed a method for mapping the affected area in the genome. This is a potentially useful addition to a battery of known methods that are currently available to study copy number variation and other structural variants in the human genome (10). Today’s arsenal of molecular diagnostic tools includes a genomic microarray with probes able to detect the level of unique genetic sequences (sequence tagged sites, STS) across all chromosomes, and this information can be obtained in some specialized hospitals. However, these relatively high-cost procedures suffer from the same shortcomings as the RNA microarray (expression data chips). Namely, the results are not conclusive, but informative, and need to be ascertained using more reliable and reproducible methods such as qPCR or fluorescent *in-situ* hybridization (FISH).

Our research could reveal genes important in pathogenesis of the WBS, we established an accurate and fast method to diagnose the disease and coarsely map the most commonly hemideleted area on chromosome 7. Presently, diagnosis of the WBS involves FISH of chromosomes with the elastin gene as a probe, which is reliable, but not entirely specific. Namely, certain rare WBS cases are misdiagnosed (in which the elastin gene was not hemideleted). Our method involves quantifying parts of chromosomal DNA in order to distinguish between their haploid and diploid content in the genome (or higher copy number variants). For this, we created an array of qPCR tests to detect hemideletions causing WBS.

Quantifying DNA in a plasmid, or in simpler genomes like in bacteria or viruses, is relatively straightforward method. However, complex genomes pose a larger problem, which the chance of cross-hybridization to similar DNA sequences. In addition, the WBSCR genetic area has its own structural complications.

The WBSCR has 3 large region-specific segmental duplications or low copy repetitive DNA elements (centromeric, medial and telomeric LCRs). Each LCR has three blocks denoted A, B and C. The block B includes genes GTF2I, NCF1 and GTF2IRD2 (or their look-alikes) that are oriented in the opposite direction in centromeric and telomeric LCRs, while the former and medial has the same orientation (Fig. 1). These are thought to be the origins of genetic instability of the region, because the area between any two duplicated sequences that is oriented in the same direction can be deleted during unequal meiotic crossingover (6). Thus, several theoretical possibilities exist for various kinds of deletions, and although the map of this area seems to be nearing completion (there is a gap of 50 Kb centromeric to WBSCR16-like gene; around 74.1 Mb), the variety of WBS deletions can be generally grouped in two, a 1.55 and less common 1.84 Mb hemideletion. The rest show smaller deletions and partial syndrome features (6). Another anomaly is the inversion of the whole region between centromeric and medial LCRs with smaller deletions in border segments (7).

Therefore, the problem, apart from mapping positions of three LCRs in each patient, lies in detecting their copy number per genome. This area can be analyzed in each patient by recent genomic CNV microarray technology, however at considerable cost, as older techniques such as pulse-field electrophoresis, bacterial artificial chromosome (BAC), P1 phage, cosmid or other molecular cloning techniques combined with fine mapping and DNA sequencing should be used to confirm the microarray results. If used to screen a large number of samples, this procedure is obviously time-consuming and cost forbidding. A simpler approach, like random DNA (shotgun) sequencing would not work in this case, because LCRs would make the mapping partially incorrect, confusing and inconclusive. A possible solution to get around these problems is to measure the copy number with reliable quantitative test such as specific-probe-detecting qPCR, as has been recently shown by others (9). However, their range for mapping was restricted to 2.5 Mb and perhaps some low copy repetitive features of the WBSCR were not anticipated to the full extent (i.e. some primers might detect multiple sites).

We therefore made an array of PCR primers to quantify several WBSCR loci, and identified specific probes to verify the origin of amplicons. In both WBS patients (Fig. 2, Tables 1 and 2), we found that the region containing WS-Dg5 and WS-ELN-2 loci was deleted on one of the chromosomes 7 (Fig. 1), demonstrating the validity of the array. These tests now permit diagnosis of the Williams-Beuren syndrome in a simpler way even before the symptoms become evident for a great majority of patients (and their parents). Why is this important?

WBS patients present a number of clinical problems already in childhood, encompassing symptoms such as cardiovascular, connective tissue and neurodevelopmental abberations. Persons with WBS have typical yet subtle cranio-facial features. Most of them have mild to moderate levels of mental retardation. Cognitive abilities such as expressive language, auditory memory and face recognition are affected. There also may be impairment in visual-spatial abilities. In contrast, musical abilities or intense interest in music and performing arts are a distinguishing feature of the phenotype (1). Likewise, people with WBS are extremely sociable, extroverted, and highly empathic in their responses to other people (14), but this sociability does not convert into more advanced social cognitive abilities (15). When psychological and social training starts early in childhood, mental abilities of WS patients seem to improve, and consequently lead to better socialization in adulthood (16). However, the WBS patients are not diagnosed early enough, because of unsuspecting, complex, and hardly recognizable morphological features of the disease.

At present, screening infants for a specific DNA deficit with a current state-of-the-art diagnostic procedure is cost forbidding, because the disease is rare and the procedure is lengthy and demanding. The incidence of a syndrome or disorder depends on the accuracy of diagnosis. The incidence of WBS is reported to be from 1 in 20.000 (17) to 1 in 50.000 (18). A recent study estimates the incidence in the region of Hong Kong to be 1 in 23.500 live births (19). An epidemiological survey from Norway suggests that the prevalence could be as high as 1 in 7.500 (20). The WBS specific qPCR test developed by the present study could permit a cost-effective, non-invasive and rapid screening of newborns (or in pre-schooling environments) that does not imply highly specialized institutions. In addition, the test also makes available prenatal diagnosis, parental testing and genetic counseling of potential parents in a faster and simpler context. While only the fetal diagnostic WBS test would be invasive (amniocentesis during pregnancy), the rest could be non-invasive, which might be an additional important advantage over the FISH method.

There are multiple research benefits (apart from the cost-effective one) that can be envisaged by the use of the here presented qPCR method. For example, about 80% of William’s syndrome patients have tooth abnormalities (11). Dental abnormalities range from enamel hypoplasia, microdontia and malocclusion, to total or partial lack of teeth in some patients (1, 11, 21). Perhaps the genes influencing certain aspects of tooth development might be found in the WBSCR. The search to find them might be helped by mapping the WBSCR using principles of the test described in this study, as hemideleted areas of patients are not the same. It is corroborated by our preliminary findings that in our partients the border area containing the GTF2I gene (its 9th intron) harboring marker D7S1870. The findings might eventually help in searching for novel treatments of patients with such dental abnormalities. By the same token, such testing might benefit clinical dentists and oral biologists in finding novel therapies in handling WBS symptoms like feeding difficulties, skeletal abnormalities, joint limitations and chronic otitis media.

In conclusion, we report here an array of qPCR-based WBS specific assays detecting hemizygous microdeletion around the elastin gene together with an increased copy number of a small border region on human chromosome 7. The test would be useful in clinical medicine and research, especially for diagnostic purposes and genetic counseling of the disease.
